# Factors associated with retinopathy of prematurity ophthalmology workload

**DOI:** 10.1038/s41372-018-0212-x

**Published:** 2018-08-31

**Authors:** Jack Jacob, Zinnia Matrix, Debra Skopec, Benjamin Ticho, Robert W. Arnold

**Affiliations:** 1 Alaska Neonatology Associates/Mednax Medical Group, Anchorage, AK USA; 2 Glacier Medical Software, Anchorage, AK USA; 3 Advocate Christ Medical Center, Oak Lawn, IL USA; 4 Alaska Children’s Eye and Strabismus, Anchorage, AK USA

## Abstract

**Objective:**

This article reports on retinopathy of prematurity (ROP) workload in the NICU related to severity of disease, gestational age at discharge, and practice variation.

**Study design:**

Data analysis on 1771 patients ≤ 30 weeks of gestation at birth from a de-identified data set of 13 NICUs.

**Results:**

There was a positive relationship between the severity of ROP and (1) the number of exams per patient, (2) the severity of ROP, and (3) postmenstrual age at discharge. The progression between the stages of ROP added to exam workload and postmenstrual age at NICU discharge. The addition of plus disease did not increase the exam burden. There was significant practice variation in the number of exams performed independent of ROP severity.

**Conclusion:**

The progression of the severity of ROP independent of plus disease, and practice variations both contribute to ROP workload. Addressing these factors could decrease ROP workload without compromising American Academy of Pediatrics (AAP) guidelines.

## Introduction

Retinopathy of prematurity (ROP) is an important cause of childhood vision loss in the United States and worldwide [1], necessitating the devotion of significant resources for screening and detection. The problem of increasing ROP examination workload has been compounded by a shortage of ophthalmologists willing to provide ROP care, the need to decrease health care costs, and the proliferation of Newborn Intensive Care Units (NICU) providing Level 2 and Level 3 care.

There have been several approaches to addressing ROP workload. Modifying the American Academy of Pediatrics ROP guidelines [2] to exclude from screening lower-risk infants could reduce the ROP workload. This approach focuses on excluding infants at higher gestational ages with low clinical risk factors [3–9]. Alternatively, telemedicine (using store and forward retinal images) reduces the frequency of on-site ophthalmologist examinations, with only those babies approaching threshold ROP transferred to a referral facility [10].

Progression of ROP severity may be related to multiple factors in NICU care management [11–13], including supplemental oxygen administration, careful monitoring and manipulation of patient oxygen saturation, and maintenance of function residual capacity (FRC) through adjustments to continuous positive airway pressure (CPAP) and noninvasive ventilation techniques [11].

Another potential avenue for addressing ROP workload may be to investigate the impact of ROP severity, progression, and practice variation between NICUs on the number of ROP exams conducted. This could provide an avenue for decreasing ROP workload without compromising current AAP guidelines. We approached this by comparing the ROP stage severity and number of ROP examinations from 13 US Level III and IV NICUs using ROP Check^©^ software [14].

## Methods

We have previously reported on a cloud-based electronic medical record (ROP Check^©^) for scheduling, tracking, and documenting ROP exams both in the inpatient and outpatient setting [14, 15]. This paper reports on an analysis of the de-identified data set from ROP Check related to ROP ophthalmology workload. The data that support the findings of this study are available from ROP Check but restrictions apply to the availability of these data, which were used under license for the current study, and so are not publicly available. Data are however available from the authors upon reasonable request and with permission of ROP Check.

We report on 1771 surviving patients in 13 NICUs in the United States who have been using the program from 2011 through 2015. The program was initiated in different units at different time periods between 2011 and 2013.

The data exclude patients who died or were transferred to another NICU not using ROP Check or referred late in their care for ROP treatment. In this paper, we report on the number of ROP exams performed, the gestational age at which babies were discharged from routine ROP detection exams, and institutional variation in these variables.

SPSS version 22 was used for statistical analyses [16]. Institutional review board (IRB) approval was obtained for the study. Analysis of variance (ANOVA) was used to analyze the relationship between the stage of ROP and number of exams conducted, and the relationship between postmenstrual age at discharge from acute ROP care and the number of exams conducted. Analysis of covariance (ANCOVA) was used to analyze the differences in the number of exams conducted between NICUs after controlling for differences in gestational age and severity of ROP. Sample size for ANOVA with 3×4 factors and an alpha of 0.05 to detect a difference of 1.0 needs *n* ≥ 40 per factor. For the latter analyses, bootstrapping was used to derive estimates of the error terms for the overall main effect and for the pairwise differences between site means. Bootstrapping is a statistical method used for estimating statistical parameters by continued resampling of an empiric sample.

## Results

### Sample characteristics

The sample consisted of 1771 prematurely born infants between 22 and 30 completed weeks of gestation. ROP was classified as no ROP, stage 1, 2, and 3 ROP. There was only one infant that had ROP beyond stage 3. The frequencies of each gestational age category, number of exams conducted at each gestational age category, and ROP severity level are shown in Table 1. The majority of patients were in the highest gestational age category (28–30 weeks), and the lowest gestational age category (22–24 weeks) represented only about 13% of patients. A majority of patients had no ROP (58.1%) and only 7% of patients were diagnosed with stage 3 ROP. There was a total of 4183 exams performed with the largest number performed in babies at 28–30 weeks of gestation (52.2%) and the lowest in the most immature infants at 22–24 weeks of gestation (21.6%). Data are presented showing the worst stage of ROP diagnosed in either eye for each progression of ROP stage from no ROP to stage 3.

**Table 1:** Frequencies of sample characteristics

Sample characteristics	Category	Frequency	Percent
Gestational age	22–24 weeks	234	13.2%
	25–27 weeks	600	33.9%
	28–30 weeks	937	52.9%
	Total	1771	100%
Number of exams	22–24 weeks	90	21.6%
	25–27 weeks	1094	26.2%
	28–30 weeks	184	52.2%
	Total	4183	100%
ROP severity	No ROP	1029	58.1%
	Stage 1	367	20.7%
	Stage 2	251	14.2%
	Stage 3	124	7.0%
	Total	1771	100%

### Tests of hypotheses

Our first assumption was that there is a positive relationship between the number of exams per infant and severity of ROP; in other words, infants with a worse disease would require more examinations. Although obvious, our intent was to quantify the effect of each severity progression. This hypothesis was tested using analysis of variance (i.e., ANOVA) to estimate the sums of squares explained by the linear component of the polynomial contrast for the four

severity levels of ROP. The positive linear relationship between the severity of ROP and the mean number of exams conducted shown in Table 2 is statistically significant (*p* *<* 0.001). The impact of the severity of ROP on the number of exams conducted is large; the linear relationship explains 51.2% of the variance in the mean number of exams per severity level. There was a progression for every stage of ROP, including progression from no ROP to stage 1 ROP. In addition, more immature infants had a greater number of exams for each stage of ROP compared to more mature infants.

**Table 2:** Mean number of exams (±std. error) by ROP level and gestational age category

ROP severity	Total	22–24 weeks	25–27 weeks	28–30 weeks
No ROP	2.3 ± 0.08	4.4 ± 0.62	3.3 ± 0.12	2.0 ± 0.06
Stage 1	4.5 ± 0.01	5.9 ± 0.24	4.9 ± 0.12	3.2 ± 0.16
Stage 2	8.0 ± 0.19	9.0 ± 0.17	7.5 ± 0.16	6.2 ± 0.36
Stage 3	11.0 ± 0.27	11.5 ± 0.21	10.7 ± 0.25	8.8 ± 0.62
All patients		7.8 ± 3.4	5.4 ± 2.9	2.3 ± 1.4

The second assumption proposed that there is a positive relationship between postmenstrual age at discharge from acute ROP care and severity of ROP. This hypothesis was tested using analysis of variance (i.e., ANOVA) to estimate the sum of squares of postmenstrual age at discharge that are explained by the linear component of the polynomial contrast for the severity of ROP levels. The positive linear relationship between ROP severity level and postmenstrual age at discharge from acute ROP care shown in Table 3 is statistically significant (*p* *<* 0.001). Moreover, the effect size is large; the linear relationship explains 30.2% of the variance in the age at discharge per severity level. Furthermore, the postmenstrual age at discharge from acute ROP care was progressively later for every progression of ROP severity, including progression from no ROP to stage 1 ROP.

**Table 3:** Mean gestational age at discharge (±std. error) by ROP level and gestational age category

ROP severity	Total	22–24 weeks	25–27 weeks	28–30 weeks
No ROP	36.0 ± 0.16	37.0 ± 3.0	36.7 ± 2.5	35.9 ± 2.0
Stage 1	38.2 ± 0.17	39.2 ± 2.9	38.2 ± 4.3	37.8 ± 3.1
Stage 2	40.5 ± 0.26	40.7 ± 4.3	40.2 ± 4.1	39.7 ± 4.1
Stage 3	44.0 ± 0.43	43.9 ± 5.1	43.3 ± 3.8	45.3 ± 4.1

The impact on the overall exam workload varied based on birth gestational age because fewer infants at 28–30 weeks of gestation progressed to stage 2 or 3 ROP compared to infants at 22–24 weeks of gestation. Table 4 shows the number of exams or exam burden added to the ROP workload with each progression of ROP for the different gestational age groups. The table also shows the additional weeks added to the time of discharge from acute ROP care. The progression from no ROP to stage 1 ROP added an additional 12% to the exam burden and 1.7 weeks to the gestational age at discharge; the progression from stage 1 to stage 2 ROP added an additional 15% to the exam burden and 1.8 weeks to the gestational age at discharge from ROP exams; progression from stage 2 to stage 3 ROP added 7.3% to the exam burden and 3.3 weeks to the gestational age at discharge from ROP exams.

**Table 4:** Additional exam burden and delay at gestational age (GA) at discharge for each progression of ROP (total exams = 4183)

ROP progression			
GA category	No ROP to stage 1	Stage 1 to stage 2	Stage 2 to stage 3
22–24 weeks			
Additional exams	77 (1.8%)	298 (7.1%)	140 (3.3%)
GA at discharge	+2.2 weeks	+1.5 weeks	+3.2 weeks
25–27 weeks			
Additional exams	298 (7.1%)	291 (7.0%)	144 (3.4%)
GA at discharge	+1.5 weeks	+2.0 weeks	+3.1 weeks
28–30 weeks			
Additional exams	132 (3.2%)	48 (1.1%)	21 (0.5%)
GA at discharge	+1.9 weeks	+1.9 weeks	+5.6 weeks

The addition of pre-plus and plus disease to the various stages of ROP had a minimal effect on the number of exams conducted and the postmenstrual age at discharge from acute ROP care (data not shown). The addition of treated babies (7.4% of infants) also did not add an additional burden on ROP exams beyond the stage of ROP.

We also investigated whether there are institutional differences in the number of ROP examinations conducted, after controlling for differences in ROP severity and gestational age. This hypothesis was tested by conducting an analysis of covariance (ANCOVA) of the number of exams by site, specifying the severity of ROP and gestational age at discharge as covariates. Bootstrapping was used to derive empirical estimates of the error terms for the overall main effect and for pairwise differences between means. The covariate-adjusted means for each site ranged from 3.7 to 4.9 with a median of 4.0. The overall effect for the site, controlling for ROP severity and gestational age at discharge was significant (*p* *<* 0.001). The covariate means fell into a spectrum with two sites that were outliers, having the highest number of exams. For infants with advanced ROP (stages 2–3), this institutional effect was most prominent for babies with gestational age of 28–30 weeks.

The relative contribution of ROP severity and progression, gestational age at birth, and institution to the number of exams was as follows: ROP severity 39.9%, gestational age 42.5%, and institution 8.2%.

## Discussion

Examination workload has been an important issue for NICUs and pediatric ophthalmologists providing ROP care. Efforts to address workload by narrowing screening guidelines, for example, by narrowing the catchment window to infants below 29 or 30 weeks of gestation, may cause some infants requiring treatment to be missed, with attendant personal and societal costs of otherwise preventable blindness. Screening algorithms such as those from Colorado and WINROP [6–8] add clinical data on a baby to enhance AAP guidelines in an attempt to decrease the number of babies screened. Such studies suffer from study setting acquisition bias. Hutchinson et al. have cautioned that screening algorithms focused on modifying AAP guidelines are not ready for widespread use without additional study [17]. Furthermore, we have previously demonstrated that current AAP screening guidelines performed exceptionally well in providing a safety net for capturing all infants, even those at low risk for severe ROP [15], adding caution to the notion of modifying AAP screening guidelines in a clinical practice setting. Finally, there has been work on a telemedicine approach to addressing ROP workload [10]. This solution, although reducing the frequency of on-site ophthalmology examinations, may actually increase the number of examinations conducted [18]. Our study contributes to the literature on ROP ophthalmology workload by investigating the contribution of gestational age, ROP severity, ROP progression, and institutional practice variation.

Our results confirm the increase in ROP exams with younger gestational age, ROP severity, and ROP progression. Although this is expected, our data quantify the contribution of each of these factors and therefore provides a foundation for a better understanding of the contributors to ROP workload. We think it is unexpected that progression from no ROP to stage 1 ROP added to ROP workload in every gestational age category. The reason for this is unknown, but further investigation could help provide strategies for decreasing ROP workload while still following AAP screening guidelines.

It was also unexpected that the addition of pre-plus and plus disease did not add to the ophthalmology workload. It is likely that infants reaching the threshold posterior to mid-zone 2 will take several weeks to reach full maturity. Many of these infants are candidates for treatment which arrests the progression of ROP, thus mitigating the need for many additional exams during the acute phase of ROP. However, such infants may have a need for longer-term pediatric ophthalmology follow-up in infancy and childhood because of other ophthalmology morbidities [19].

We demonstrated institutional variation in the number of exams independent of the severity of ROP and gestational age at birth. This institutional variation raises several possibilities. AAP re-examination guidelines have some degree of latitude for the ophthalmologist. These guidelines set clear outer limits for when exams are conducted in order not to miss treatment-warranted ROP. These AAP guidelines are incorporated into ROP Check^©^ decision support in order to prevent missing treatment-warranted ROP. This allows an ophthalmologist to choose more frequent exams which could contribute to this institutional variation. Our observation that each progression of ROP (including minor progression from no ROP to stage 1 ROP) was accompanied by a progressive delay in postmenstrual age at discharge from acute ROP care suggests that exams are extending longer rather than at more frequent intervals. This could suggest delayed retinal maturity even with mild ROP. This deserves further investigation. Finally, NICU performance improvements in ROP care could lead to reduction in ROP progression, with attendant beneficial decrease in required ROP examinations.

A fundamental concept in quality improvement work is to address provider-based practice variation that increases health care costs without attendant improvement in quality of care [20]. Our data show that some NICUs performed significantly fewer ROP examinations when corrected for ROP severity than other institutions. In our cohort of babies, there were no infants that had a delay in treatment-warranted ROP resulting in a poor outcome, suggesting that an effort to decrease provider-based variation would be unlikely to have an adverse effect on ROP outcome.

Our personal experience in working with many NICUs across the United States is that pediatric ophthalmologists have generally taken a passive role in addressing ROP care practice in the NICU. In addition, neonatologists have not incorporated pediatric ophthalmology into their quality improvement efforts related to ROP care. We suggest that there is a need for neonatologists to actively collaborate with pediatric ophthalmologists in aspects of NICU care that affect ROP severity, practice variation, and ophthalmology workload.

Much of the focus of quality improvement efforts in NICUs in ROP care has been the prevention of blindness and treatment-warranted ROP. Our results suggest that to address ROP workload, prevention of treatment-warranted ROP should not be the only goal. Sub-treatment-level disease progression requires increased frequency of ROP examinations and adds substantially to the ophthalmology workload burden. This issue should be considered by neonatologists and NICU personnel involved in quality improvement work.

Another issue that is important in the provision of ROP care relates to the frequency of babies being discharged from NICUs prior to achieving retinal maturity. The extent of this practice is unknown. Our study showed a delay in gestational age at discharge from active ROP care as the severity of ROP increased. Incomplete retinal maturity in an infant otherwise ready for discharge from the NICU places pressures on neonatologists for discharge. Our previous study showed that outpatient ROP care is common in some institutions and that many such infants have advanced ROP and frequently have missed or delayed appointments, making this a high-risk practice for ophthalmologists, neonatologists, NICUs, and primary care pediatricians [15]. Therefore, discharge from NICUs for babies who have not achieved retinal maturity is another potential productive area for quality improvement and collaboration between neonatologists and ophthalmologists.

Our study has several limitations. First, the study may not be representative of practices across the United States. Institutions and pediatric ophthalmologists electing to use ROP Check^©^ represent a select group that may have a greater focus on ROP care. Second, detailed clinical information on patients is not available within ROP Check, so our ability to look at clinical factors that affect ROP severity is limited. Nevertheless, our study provides additional avenues for addressing the issue of ROP workload.
